# Recent advances on cyclodepsipeptides: biologically active compounds for drug research

**DOI:** 10.3389/fmicb.2023.1276928

**Published:** 2023-10-02

**Authors:** Si-Xuan Liu, Si-Yi Ou-Yang, Yong-Fu Lu, Chun-Lin Guo, Si-Yang Dai, Chang Li, Tian-Yi Yu, Yue-Hu Pei

**Affiliations:** ^1^Department of Medicinal Chemistry and Natural Medicine Chemistry, College of Pharmacy, Harbin Medical University, Harbin, China; ^2^Key Laboratory of Gut Microbiota and Pharmacogenomics of Heilongjiang Province, College of Pharmacy, Harbin Medical University, Harbin, China; ^3^The Third Affiliated Hospital of Heilongjiang University of Traditional Chinese Medicine, Harbin, China

**Keywords:** cyclodepsipeptide, fungi, bacteria, antimicrobial, biological activity

## Abstract

Cyclodepsipeptides are a large family of peptide-related natural products consisting of hydroxy and amino acids linked by amide and ester bonds. A number of cyclodepsipeptides have been isolated and characterized from fungi and bacteria. Most of them showed antitumor, antifungal, antiviral, antimalarial, and antitrypanosomal properties. Herein, this review summarizes the recent literatures (2010–2022) on the progress of cyclodepsipeptides from fungi and bacteria except for those of marine origin, in order to enrich our knowledge about their structural features and biological sources.

## Introduction

Cyclodepsipeptides are an important group of polypeptides, which contain one or more amino acids replaced by a hydroxy acid, resulting in at least one ester bond in the core ring structure (Lemmens-Gruber et al., 2009; Buckton et al., 2021). Cyclodepsipeptides exhibit a broad spectrum of biological activities including antitumor, antifungal, antiviral, antimalarial, and antitrypanosomal activities (Moore, 1996; Nihei et al., 1998; Du et al., 2014). Due to their unique structural and biological properties, cyclodepsipeptides have emerged as promising lead structures for crop protection and human and veterinary medicine (Sivanathan and Scherkenbeck, 2014).

**Figure 1:**
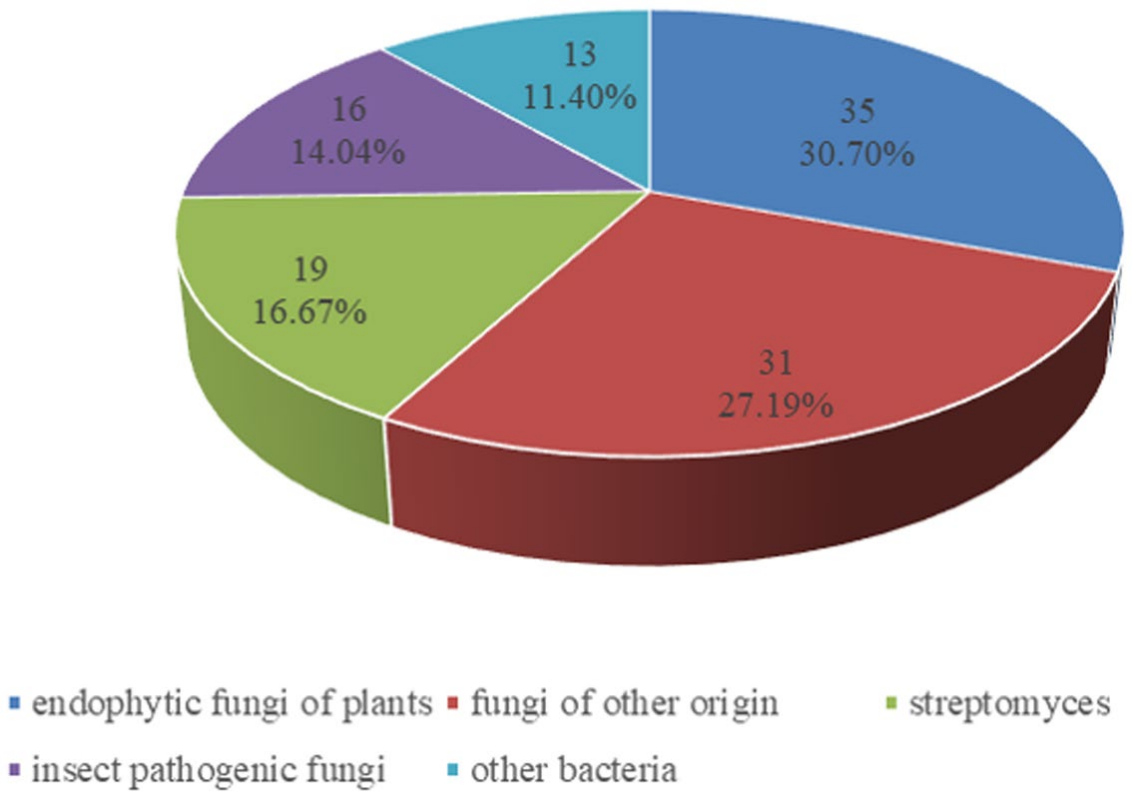
Percentage distribution of cyclodepsipeptides from fungi and bacteria.

The dominant sources of cyclodepsipeptides are fermentations of various fungi and bacteria. In addition, abundant cyclodepsipeptides have been isolated from algae, plants and marine organism (Sivanathan and Scherkenbeck, 2014; Negi et al., 2017). Because several reports have summarized the progress of the marine cyclodepsipeptides (Zeng et al., 2023), our review focused on the recent advances of cyclodepsipeptides from bacteria and fungi from plants, insects or soil except for marine organism. As a result, from 2010 to the present, 114 cyclodepsipeptides have been isolated and identified through an extensive literature search, including Web of Science, SciFinder, and PubChem tools. Included in the list of search terms were “cyclodepsipeptides,” “endophytic fungi,” and “insect pathogenic fungi” as well as “streptomyces,” “fungi,” “bacteria.” As shown in Figure 1, the major producers of cyclodepsipeptides are endophytic fungi of plants, which made up 30.70%, followed by fungi of other origin (27.19%), streptomyces (16.67%), insect pathogenic fungi (14.04%), and other bacteria (11.40%).

## Biologically active cyclodepsipeptides

### Cyclodepsipeptides from endophytic fungi of plants

**Figure 2:**
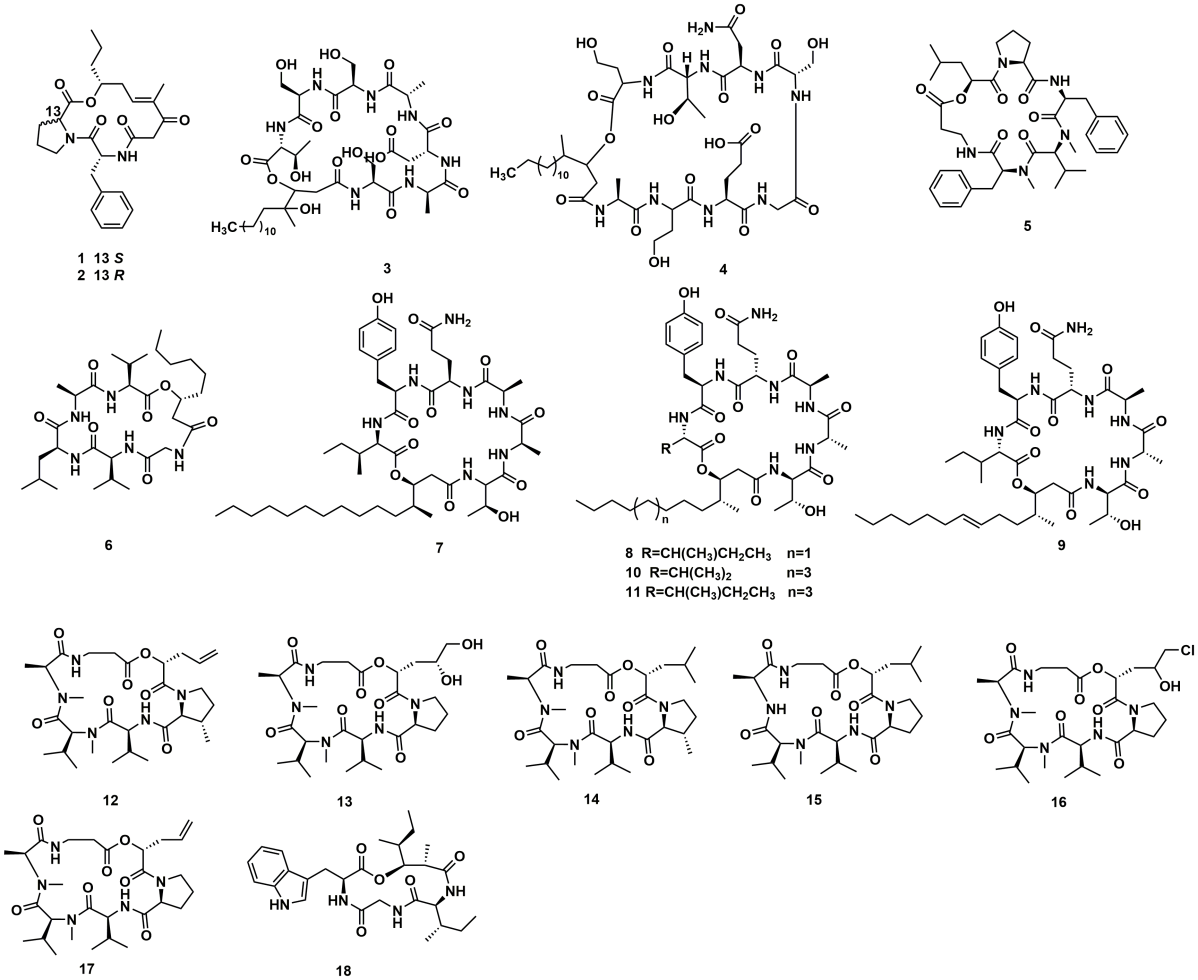
Chemical structures of CDPs **1**–**18**.

The endophytic fungi from *Fusarium and Tricholderma* genus were the main producers of cyclodepsipeptides. Chemical investigation on the fungus *Sarocladium kiliense* HDN11-112 from mangroves, led to the characterization of saroclide A (**1**) and saroclide B (**2**), two cyclic depsipeptides with 7-hydroxy-4-methyl-3-oxodec-4-enoic acid (HMODA) unit. They were a couple of epimerides with different L-and D-Pro configurations of the proline units. Saroclide A (**1**) exhibited a lowering blood lipid effect, while saroclide A (**1**) and B (**2**) were invalid for four pathogenic microorganisms and five cancer cell lines (Guo et al., 2018). Using a genome-wide *Candida albicans* fitness test, a new cyclodepsipeptide phaeofungin (**3**) containing a β,γ-dihydroxy-γ-methylhexadecanoic acid (DHMHDA) unit and seven amino acids, was isolated from *Phaeosphaeria* sp. Using the same method, another structurally different cyclodepsipeptide, phomafungin (**4**), was isolated from *Phoma* sp. **3** showed good antifungal activity to *Aspergillus fumigatus* with the MIC value at 8–16 μg/mL and *Trichophyton mentagrophytes* with the MIC value at 4 μg/mL (Singh et al., 2013). Isaridins A (**5**) and B (**6**) were isolated from *Beauveria* sp. Lr89, which was isolated from the roots of *Maytenus hookeri* Loes (Li et al., 2011). Fusaripeptide A (**7**), a novel cyclodepsipeptide, obtained from the culture of the plant endophytic fungus *Fusarium* sp. **7** showed significant antifungal activity against *A. fumigates, Candida glabrata, Candida albicans* and *Candida krusei* (IC_50_ 0.11–0.24 μM). In addition, it exhibited potent anti-malarial activity against *Plasmodium falciparum* (IC_50_ 0.34 μM) (Ibrahim et al., 2018). W493 C (**8**) and W493 D (**9**), two novel cyclic depsipeptides, as well as two known cyclic depsipeptides, W493 A (**10**) and W493 B (**11**), were isolated from the mangrove plant *Ceriops tagal* endophytic fungus *Fusarium* sp. **8** and **9** showed antifungal activity toward *Cladosporium cladosporiodes* (Lv et al., 2015). Bioassay-guided isolation and purification yielded four new cyclodepsipeptides, trichodestruxins A (**12**), B (**13**), C (**14**) and D (**15**), as well as two known cyclodepsipeptides, destruxin E2 chlorohydrin (**16**) and destruxin A2 (**17**), isolated from the fungus *Trichoderma harzianum*. **14** and **16** were evaluated for their cytotoxicity against HT-29, A549, and P388 cell lines with IC_50_ values at 0.7–10.3 μM (Liu et al., 2020; Table 1). Chaetomiamide A (**18**), a rare cyclodepsipeptide, was isolated from the fungus *Chaetomium* sp. from the root of *Cymbidium goeringii*, it also exhibited weak cytotoxicity toward HL-60 cell line with IC_50_ values of 35.2 μM (Wang et al., 2017; Figure 2).

**Table 1:** The inhibitory effects of CDPs on different cancer cell lines.

Compound	Cells	IC_50_ (μM)	References	Compound	Cells	IC_50_ (μM)	References
12	HT-29	7.8 ± 0.1	Liu et al. (2020)	27	Huh-7	3.3 ± 0.3	Wang et al. (2022)
	A549	15.6 ± 0.1			MRMT-1	2.8 ± 0.2	
	P388	17.0 ± 0.3			HepG-2	1.4 ± 0.3	
13	HT-29	16.7 ± 0.2	Liu et al. (2020)	28	Huh-7	9.1 ± 1.0	Wang et al. (2022)
	A549	8.8 ± 0.1			MRMT-1	6.8 ± 0.5	
	P388	19.1 ± 0.2			HepG-2	3.3 ± 0.4	
14	HT-29	3.4 ± 0.1	Liu et al. (2020)	29	Huh-7	6.2 ± 0.5	Wang et al. (2022)
	A549	10.3 ± 0.3			MRMT-1	2.7 ± 0.2	
	P388	8.4 ± 0.1			HepG-2	2.8 ± 0.2	
15	A549	17.5 ± 0.1	Liu et al. (2020)	30	Huh-7	>50	Wang et al. (2022)
16	HT-29	0.7 ± 0.1	Liu et al. (2020)		MRMT-1	>50	
	A549	4.9 ± 0.2			HepG-2	14.1 ± 3.4	
	P388	3.7 ± 0.1		39	HepG-2	>50	Wang et al. (2014)
17	HT-29	9.3 ± 0.2	Liu et al. (2020)		HepG-2/ADM	>50	
	A549	13.1 ± 0.1		40	HepG-2	13.67 ± 2.59	Wang et al. (2014)
	P388	12.5 ± 0.2			HepG-2/ADM	14.48 ± 1.68	
18	HL-60	35.2	Wang et al. (2017)	41	HepG-2	5.04 ± 0.20	Wang et al. (2014)
23	Huh-7	4.3 ± 1.2	Wang et al. (2022)		HepG-2/ADM	2.67 ± 0.09	
	MRMT-1	8.7 ± 0.2		42	HepG-2	2.81 ± 0.86	Wang et al. (2014)
	HepG-2	1.0 ± 0.1			HepG-2/ADM	2.93 ± 0.15	
24	Huh-7	>50	Wang et al. (2022)	61	HeLa KB3.1	28	Helaly et al. (2018)
	MRMT-1	>50		82	BT-549	2.5	Luo et al. (2022)
	HepG-2	20.1 ± 11.4		104	HCT-116	0.11	Plaza et al. (2012)
25	Huh-7	30.3 ± 7.2	Wang et al. (2022)				
	MRMT-1	17.3 ± 1.1					
	HepG-2	15.4 ± 4.2					
26	Huh-7	46.2 ± 2.5	Wang et al. (2022)				
	MRMT-1	>50					
	HepG-2	10.7 ± 7.7					

IC_50_, concentration required for 50% inhibition.

**Figure 3:**
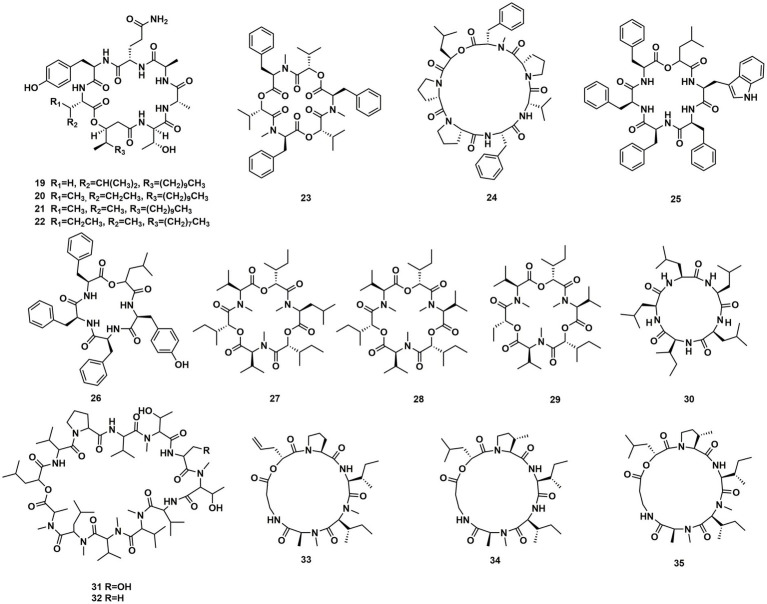
Chemical structures of CDPs **19**–**35**.

A *Celtis sinensis*-derived *Fusarium* sp. HU0174, produced acuminatums A–D (**19**–**22**), as well as beauvericin (**23**). Acuminatum D (**22**) was a novel cyclic depsipeptide. **19**–**22** exhibited potent inhibitory activity against *Penicillium digitatum* and *Curvularia lunata* using the AGAR disc diffusion methods (Li et al., 2021). Cultivation of the plant endophytic fungus *Fusarium* sp. led to the isolation of four novel compounds, fusarihexins C (**24**), D (**25**), E (**26**), and enniatin Q (**27**), together with the known beauvericin (**23**), MK1688 (**28**), enniatin I (**29**), viscumamide (**30**). The cytotoxic activities of these compounds were tested using MRMT-1, HepG-2, and Huh-7 cell lines, respectively (Table 1). **27**–**29**, and **23** exhibited strong cytotoxic activities (Wang et al., 2022; Figure 3). Two depsipeptides named xylariaceins A–B (**31**–**32**) were isolated and identified from endophytes *Xylariaceae* BSNB-0294. Both xylariaceins A (**31**) and B (**32**) inhibited the growth of *Fusarium oxysporum* (Barthélemy et al., 2021). In addition, three cyclohexadepsipeptides, destruxin A4 (**33**), trichomide B (**34**) and homodestcardin (**35**), isolated from the endophytic fungus *Fusarium chlamydosporum*, were found to be lethal to brine shrimp, **33** showed significant activity with LD_50_ at 2.78 μg/mL. It was even better than the positive control (7.75 μg /mL) (Wang et al., 2020).

**Figure 4:**
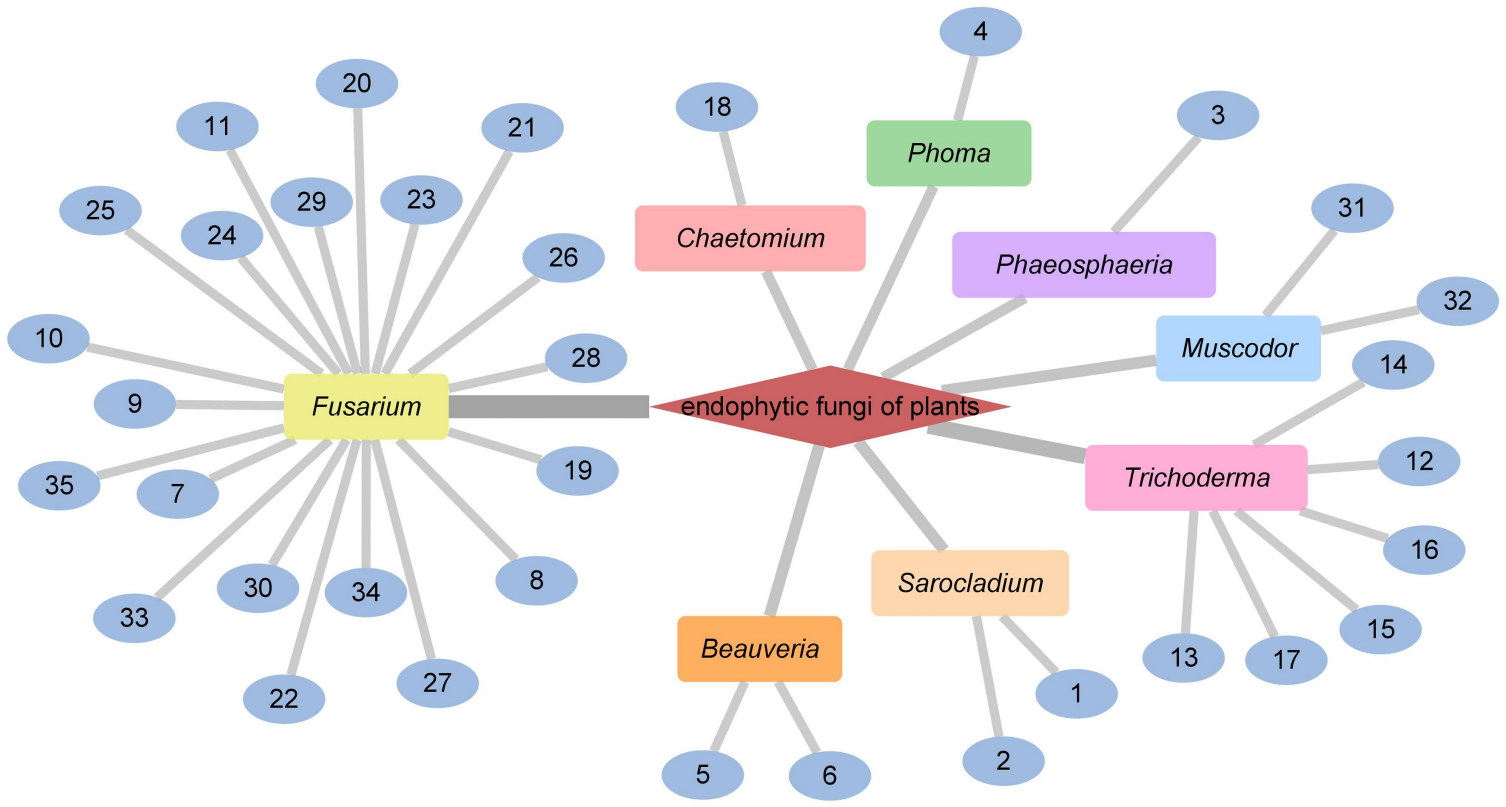
CDPs from endophytic fungi of plants.

In conclusion, the genus *Fusarium* and *Trichoderma* were the main producers of cyclodepsipeptides in endophytic fungi. In addition, the genus *Beauveria*, *Sarocladium*, *Muscodor*, *Chaetomium*, *Phoma*, *Phaeosphaeria* could produce diverse cyclodepsipeptides as well (Figure 4).

### Cyclodepsipeptides from insect pathogenic fungi

**Figure 5:**
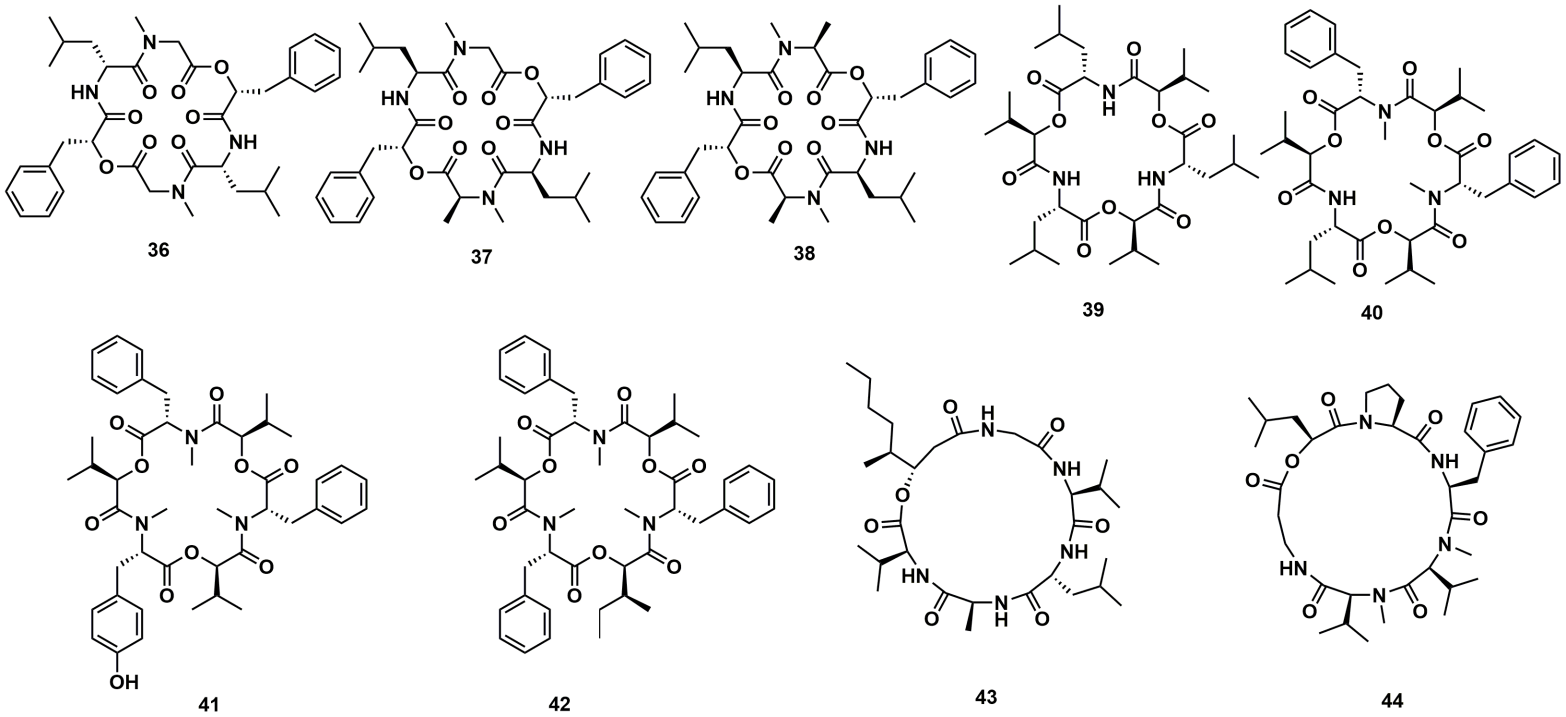
Chemical structures of CDPs **36**–**44**.

*Cordyceps cardinalis* NBRC 103832, the insect pathogenic fungus, yielded a class of new depsipeptides, cardinalisamides A (**36**), B (**37**), C (**38**). The bioactivity results indicated that **36**–**38** exhibited *in vitro* antitrypanosomal activity against *Trypanosoma brucei brucei* with IC_50_ values of 8.56, 8.65 and 8.63 μg/mL, respectively (Umeyama et al., 2014). Cordycecin A (**39**), a new cyclodepsipeptide, together with beauvericin E (**40**), beauvericin J (**41**), beauvericin (**23**) and beauvericin A (**42**), were isolated from the fungus *Cordyceps cicadae*, which was a fungus parasitic on the larvae of *Cicada flammat* as the host insect (Wang et al., 2014). Compounds **23**, **40–42** exhibited cytotoxicity toward HepG2 and HepG2/ADM cells with IC_50_ values at 2.40–14.48 μM. The insect pathogenic fungus *Beauveria felina* yielded iso-isariin B (**43**) and isaridin E (**44**) (Langenfeld et al., 2011; Figure 5).

**Figure 6:**
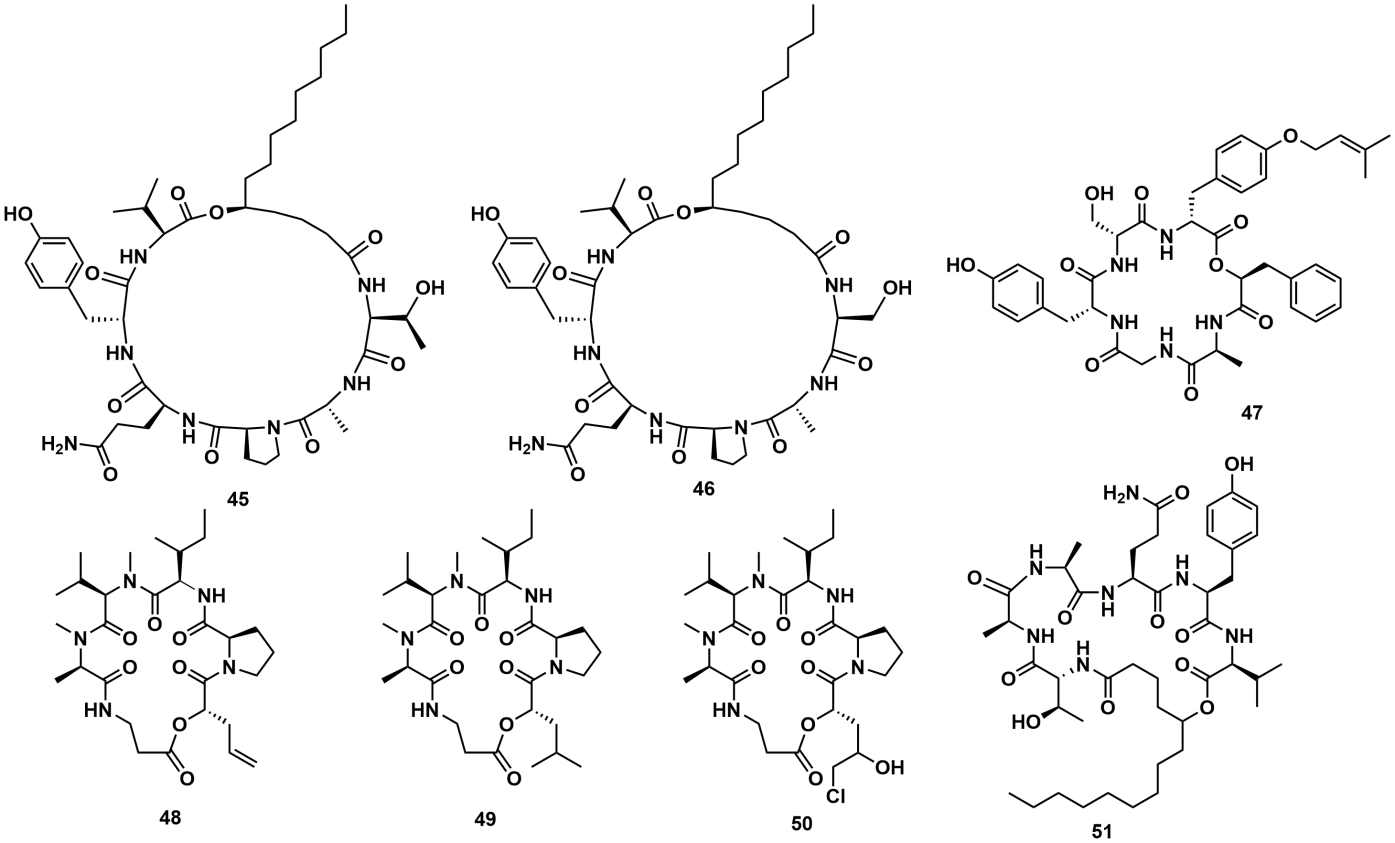
Chemical structures of CDPs **45**–**51**.

**Figure 7:**
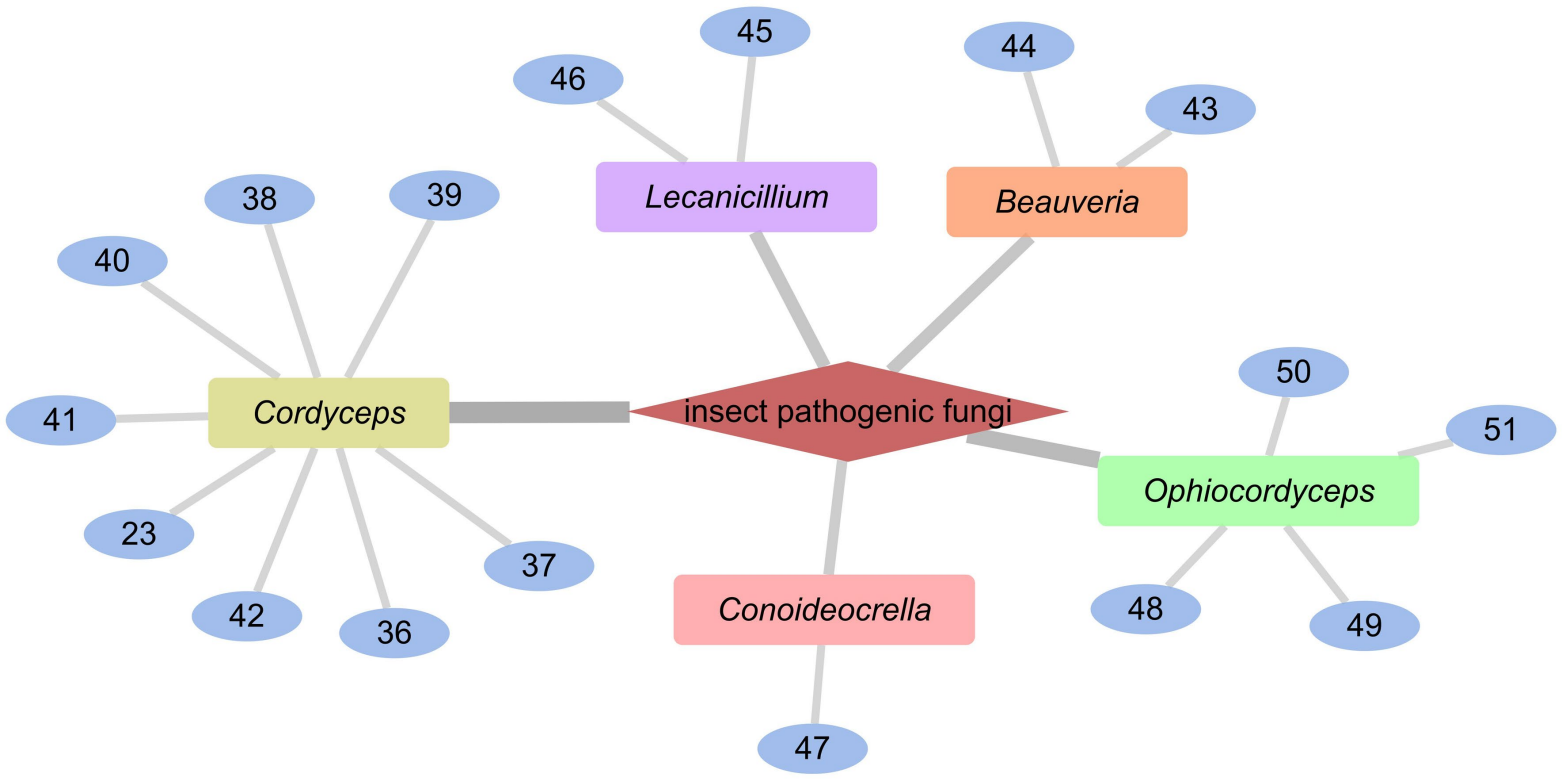
CDPs from insect pathogenic fungi.

Verlamelins A (**45**) and B (**46**) were isolated from insect pathogenic fungus *Lecanicillium* sp. HF627. **45** showed broad antifungal activity against plant pathogenic fungi (Ishidoh et al., 2014). Conoideocrellide A (**47**) was a new cyclodepsipeptide isolated from the entomopathogenic fungus *Conoideocrella. tenuis* BCC 18627. Unfortunately, tests showed that **47** had no biological activity in protocols for antiplasmodial and antiviral properties as well as cytotoxicity against a number of cancer cell lines (Isaka et al., 2011). The isolation of entomopathogenic fungus *Ophiocordyceps coccidiicola* NBRC 100683 mutant strain IU-3 provided three cyclodepsipeptides, destruxins A (**48**), B (**49**), and destruxin E chlorohydrin (**50**). The *in vitro* antitrypanosomal activity exhibited that the IC_50_ values for **48**–**50** against *T. b. brucei* GUTat3.1 were 0.33, 0.16 and 0.061 μg/mL (Ganaha et al., 2016). The entomopathogenic fungus *Ophiocordyceps communis* BCC 16475 yielded a new cyclodepsipeptide, cordycommunin (**51**). **51** was demonstrated to have growth inhibitory effect on *Mycobacterium tuberculosis* H37Ra (MIC 15 μM) (Haritakun et al., 2010; Figures 6, 7; Table 2).

**Table 2:** The anti-fungal or anti-bacteria effects of CDPs.

Compound	Strains	MIC/ IC_50_	References
3	*Candida albicans*	^a^ 16–32 μg/mL	Singh et al. (2013)
	*Aspergillus fumigatus*	^a^ 8–16 μg/mL	
	*Trichophyton mentagrophytes*	^a^ 4 μg/mL	
7	*Candida albicans*	^b^ 0.11 μM	Ibrahim et al. (2018)
	*Candida glabrata*	^b^ 0.24 μM	
	*Candida krusei*	^b^ 0.19 μM	
	*Aspergillus fumigates*	^b^ 0.14 μM	
51	*Mycobacterium tuberculosis* H37Ra	^b^ 15 μM	Haritakun et al. (2010)
62	*Bacillus subtilis*	^b^ 12.5 μM	Liang et al. (2021)
	*Alternaria solan*	^b^ 15.6 μM	
85	*Mycobacterium tuberculosis* H37Rv	^a^ 2.5 μg/mL	Liu et al. (2019)
90	*Bacillus subtilis* E168	^a^ 8 μg/mL	Bracegirdle et al. (2021)
91	*Bacillus subtilis* E168	^a^ 16 μg/mL	Bracegirdle et al. (2021)
92	*Bacillus subtilis* E168	^a^ 32 μg/mL	Bracegirdle et al. (2021)
95	*Mycobacterium bovis* bacille Calmette–Guérin (BCG)	^a^ 44.4 μM	Liu et al. (2016)
101	*Mycobacterium smegmatis*	^c^ 2 μg/mL	Dardić et al. (2017)
102	*Mycobacterium tuberculosis* mc^2^ 6,230 strains	^a^ 82.8 μg/mL	Shin et al. (2021)
106	*Candida albicans*	^b^ 73 nM	Hoffmann et al. (2015)

^a^MIC, minimal inhibitory concentration; ^b^IC_50_, concentration required for 50% inhibition; ^c^IC_80_, concentration required for 80% inhibition.

### Cyclodepsipeptides from fungi of other origin

**Figure 8:**
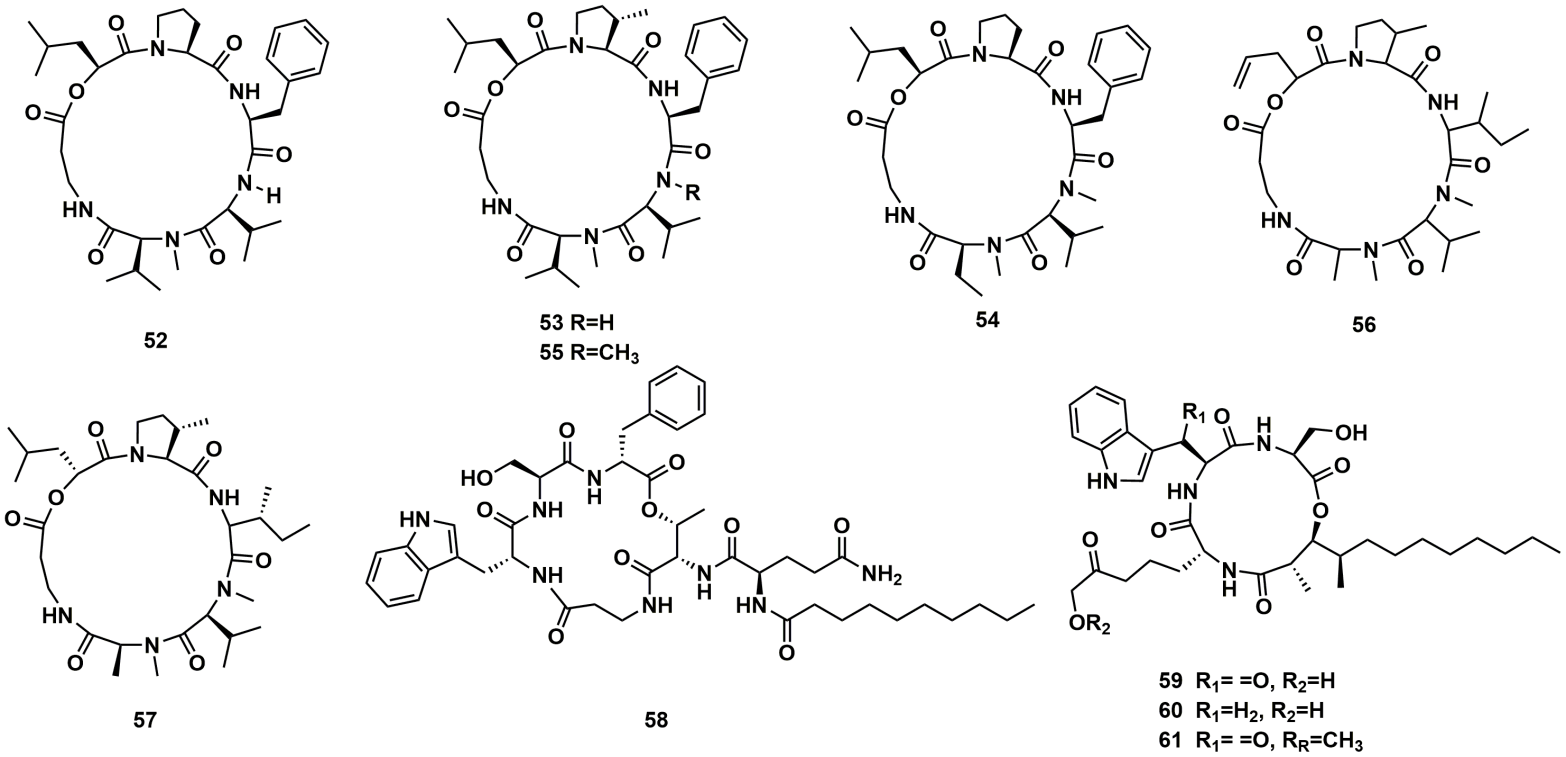
Chemical structures of CDPs **52**–**61**.

Desmethylisaridin E (**52**), desmethylisaridin C2 (**53**), isaridin *F* (**54**) and isaridin E (**44**), isaridin C2 (**55**), destruxin A (**48**), resetoxin B (**56**), and roseocardin (**57**) were collected from hefilamentous fungus *Beauveria felina*. On the elastase release in neutrophils and the FMLP-induced superoxide anion generation, **52** and **53** showed specific inhibitory action. Compound **52** demonstrated particular inhibition on superoxide anion generation with an IC_50_ at 10.0 μM, while **53** exhibited specific activity on elastase release with an IC_50_ at 10.01 μM (Chung et al., 2013). A fungus linked with nematodes that shared affinities with the genus *Ophiosphaerella,* produced ophiotine (**58**), along with two new derivatives arthrichitin (**59**) and arthrichitin B (**60**) as well as one known compound arthrichitin C (**61**). Only **61** exhibited a low level of cytotoxicity, with an IC_50_ value of 28 μM against HeLa cells KB3.1 (Helaly et al., 2018; Figure 8).

**Figure 9:**
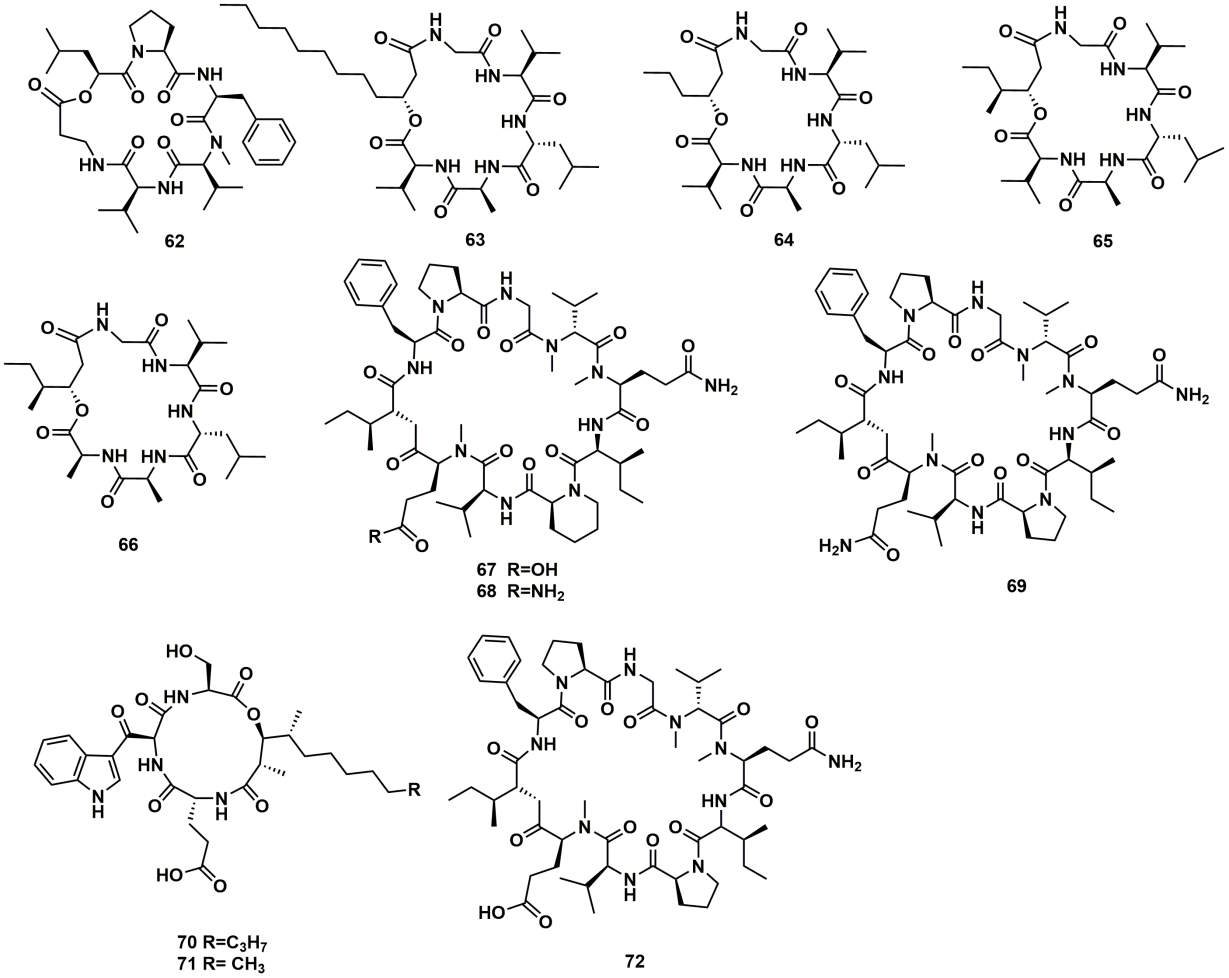
Chemical structures of CDPs **62**–**72**.

A new cyclodepsipeptide isaridin H (**62**), as well as known compounds isariin A (**63**), isariin E (**64**), nodupetide (**65**), iso-isariin D (**66**) and iso-isariin B (**43**) isaridin E (**44**), desmethylisaridin E (**52**) were identified from *Amphichorda guana.* Based on the bioassay, the novel cyclodepsipeptide isaridin H (**62**) exhibited suppression of *Bacillus subtilis* at 12.5 μM and displayed potent antifungal activities toward *Alternaria solan* (IC_50_ 15.6 μM) (Liang et al., 2021). A new class of cyclodepsipeptides SCH 217048 (**67**), SCH 218157 (**68**), together with pleosporin A (**69**) were collected from an elephant dung fungus BCC 7069. With IC_50_ values of 1.6, 6.4, and 1.6 µg/mL, respectively, all three compounds demonstrated antimalarial efficacy against *P. falciparum* K1 (Isaka et al., 2014). Artrichitin (**70**) and lipopeptide 15G256ε (**71**) were isolated from freshwater ascomycetes (*Delitschia* sp.). Under hypoxic conditions, the African American prostate cancer cell line (E006AA-hT) was resistant to the antiproliferative effects of both **70** and **71** (Rivera-Chávez et al., 2019). Two known cyclodepsipeptides named SCH 378161 (**72**) and SCH 217048 (**67**) were isolated from organic extracts of axenic *Clohesyomyces aquaticus* (Dothideomycetes) (El-Elimat et al., 2017; Figure 9).

**Figure 10:**
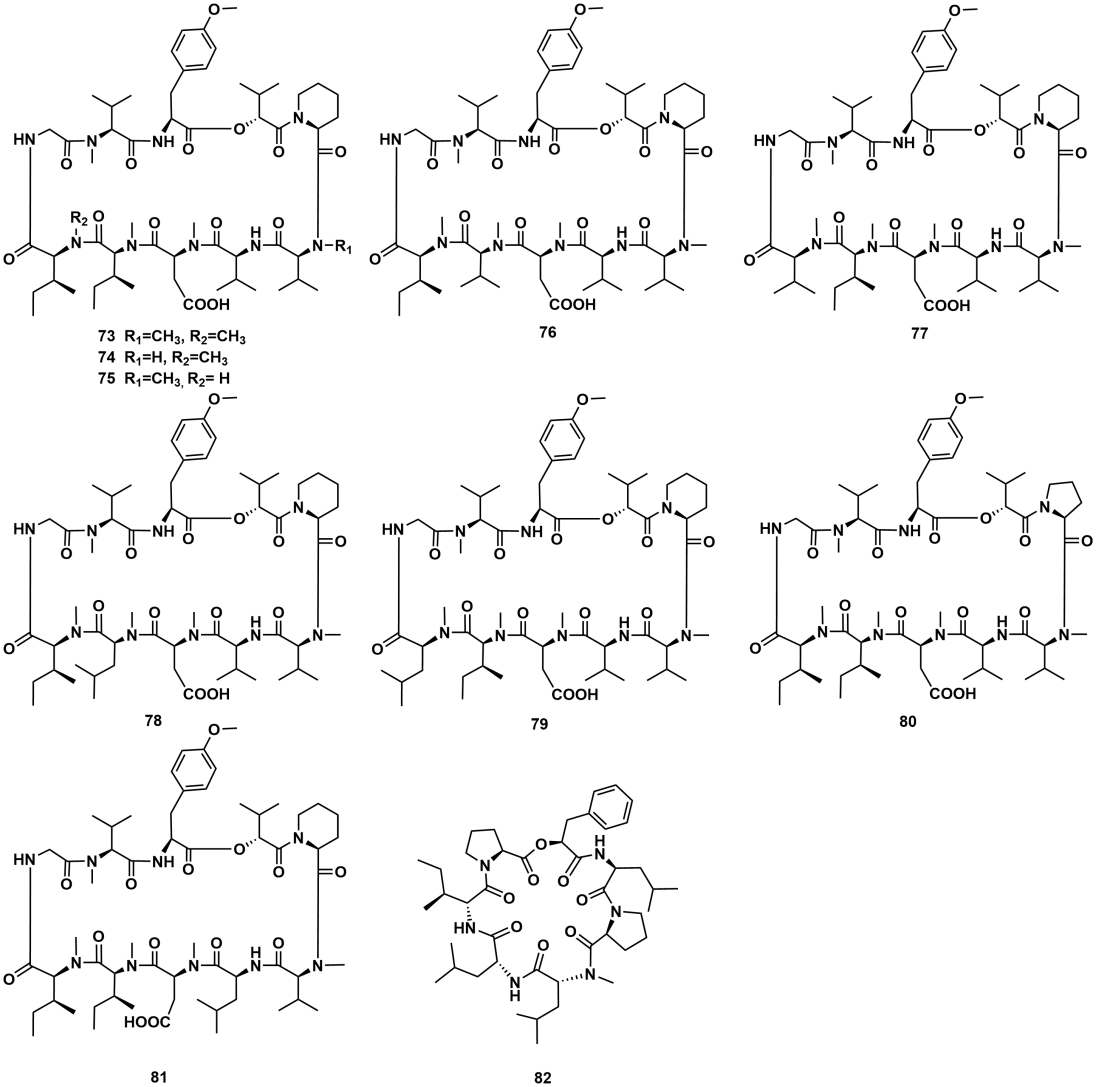
Chemical structures of CDPs **73**–**82**.

**Figure 11:**
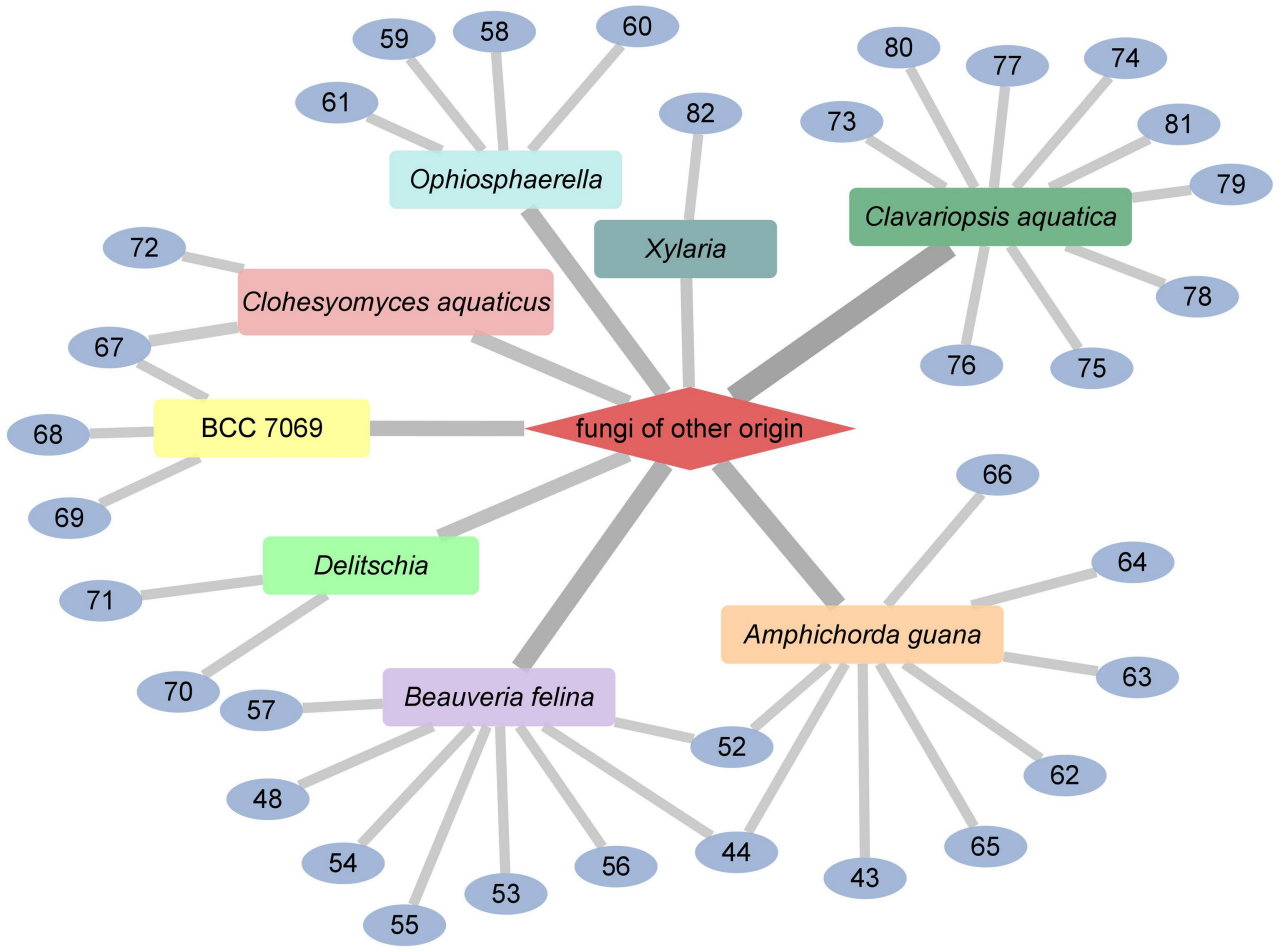
CDPs from fungi of other origin.

The cyclic depsipeptides clavariopsins A–I (**73–81**) were found through fractions of the extract of the aquatic hyphomycete *Clavariopsis aquatica*. They provided significant or moderate antifungal activity toward primarily multihost plant pathogens (Soe et al., 2019). Xylaroamide A (**82**), a cyclic depsipeptide, was isolated from an endolichenic *Xylaria* sp. Following a series of biological tests, it was discovered that **82** had an IC_50_ value of 2.5 μM and had the most powerful impact on the human triple-negative epithelial breast cancer cell line, BT-549. In addition, **82** strongly induced cell cycle arrest in G0/G1 phase in BT-549 cells by using cycle distribution analysis (Luo et al., 2022; Figures 10, 11).

### Cyclodepsipeptides from streptomyces

**Figure 12:**
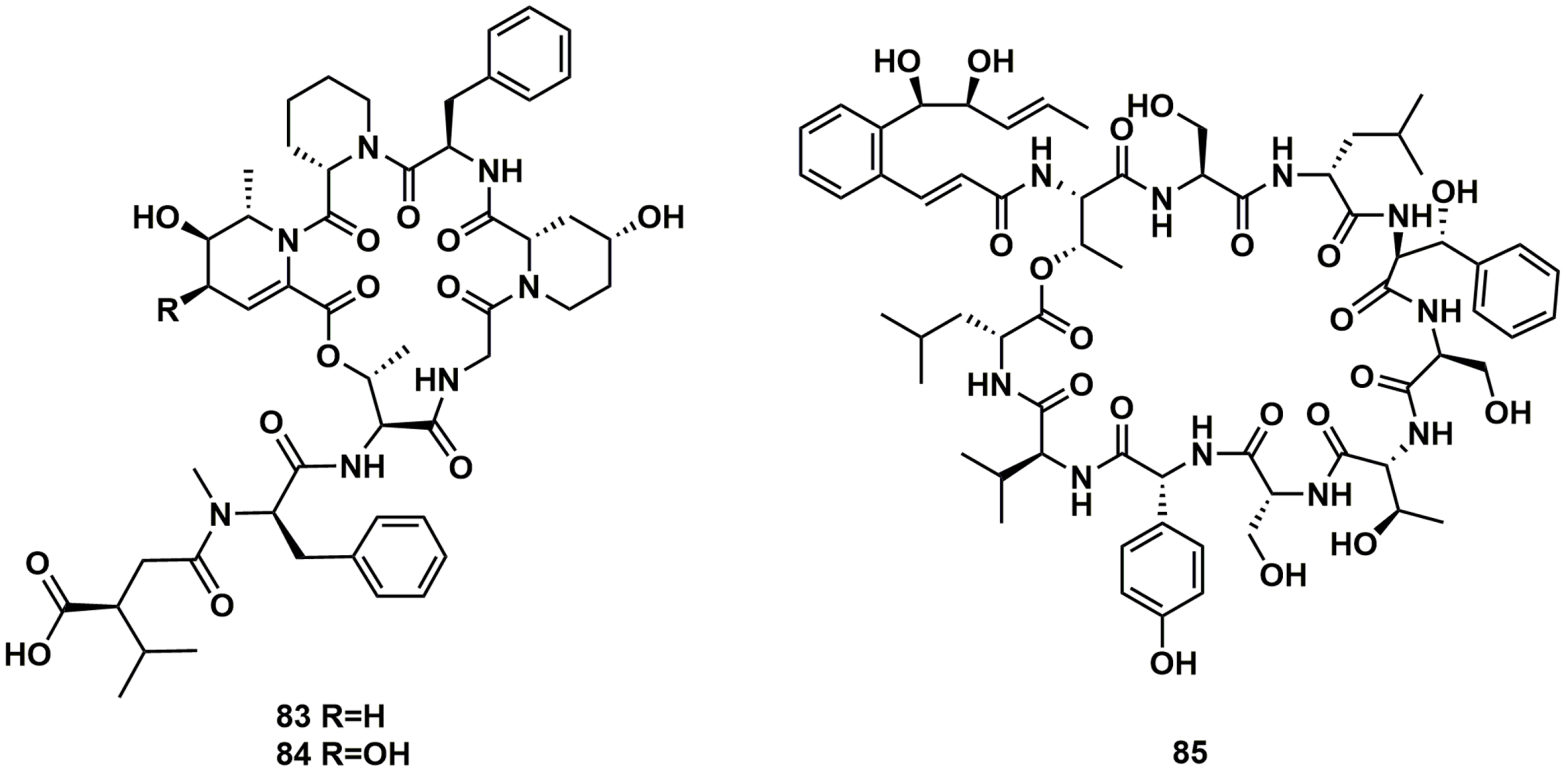
Chemical structures of CDPs **83**–**85**.

Ulleungamides A (**83**) and B (**84**), two new cyclic depsipeptides, were found in terrestrial cultured strains *Streptomyces* sp. Only *Staphylococcus aureus* and *Salmonella typhimurium* showed growth inhibition by ulleungamide A (**83**) without cytotoxicity. Besides, Comparing **83** to **84**, the selective antibacterial activity showed that the hydroxy group at position C-4 significantly lowered the activity (Son et al., 2015). Atrovimycin (**85**), featuring a novel vicinal-hydroxylated cinnamic acylchain, was a cyclodepsipeptide separated from *Streptomyces atrovirens* LQ13. A series of biological assays revealed that **85** showed a significant activity toward *F. oxysporum* and antitubercular activity against *Mycobacterium tuberculosis* H37Rv both *in vitro* (with MIC of 2.5 μg/mL) and *in vivo* (Liu et al., 2019; Figure 12).

**Figure 13:**
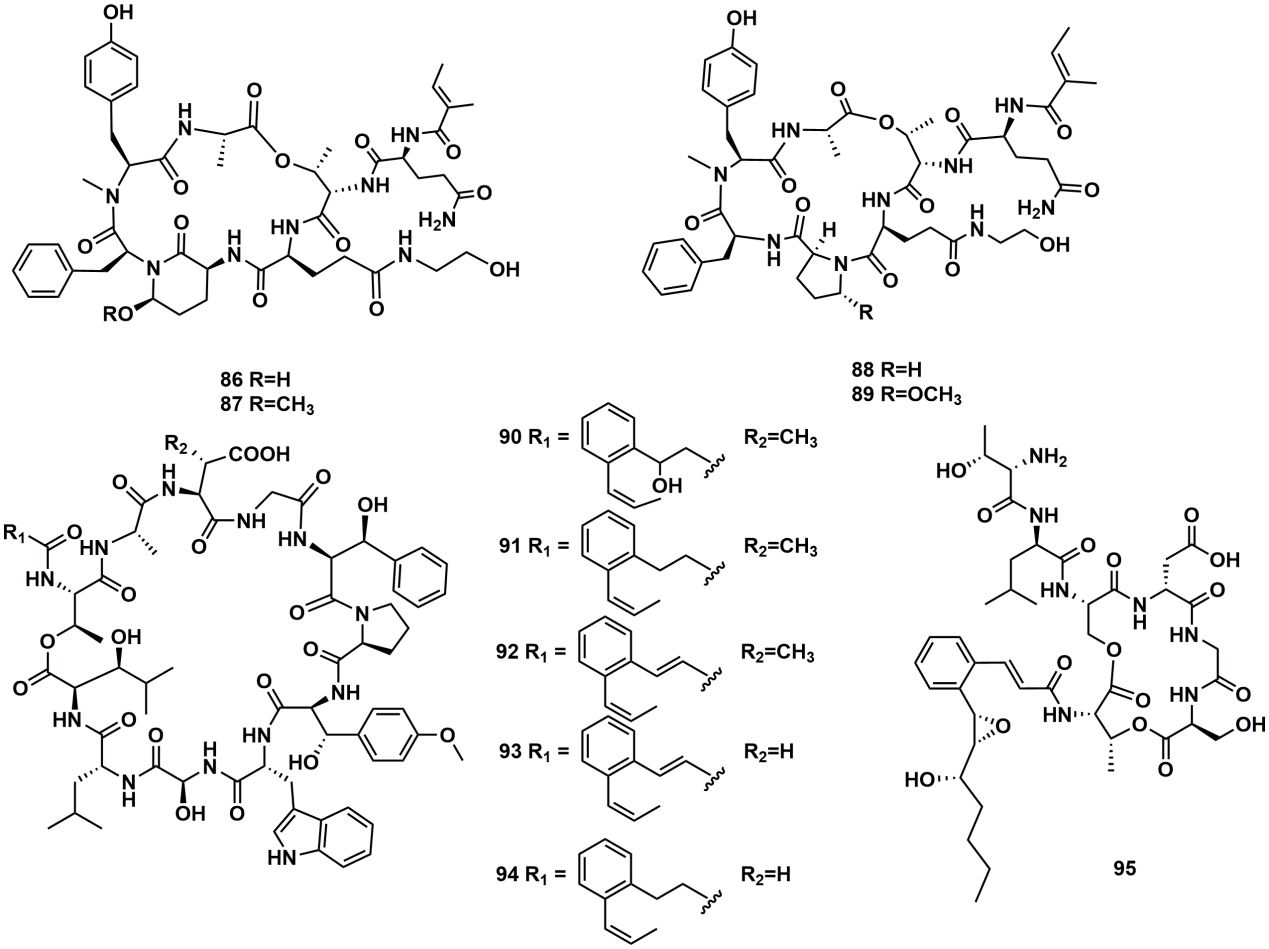
Chemical structures of CDPs **86**–**95**.

From cultures of the soil-derived *Streptomyces* sp., dinghupeptins A–D (**86–89**) were isolated. The 3-amino-6-hydroxypiperidone unit-containing compounds **86** and **87** demonstrated selective chymotrypsin inhibitory action, with IC_50_ values of 2.1 and 1.1 μM, respectively (Yang et al., 2018). Skyllamycins D (**90**) and E (**91**) were isolated from Lichen-sourced *Streptomyces* sp. KY11784, as well as three known ones skyllamycins A − C (**92**–**94**). Antibacterial tests showed that (Table 2), in comparison to previously reported congeners, skyllamycin D (**90**) had better activity against *Bacillus. subtilis* E168 (Bracegirdle et al., 2021). NC-1 (**95**), a cyclodepsipeptide containing a cinnamoyl side chain, was discovered in *Streptomyces* sp. FXJ1.172 that originated in red soil. **95** was evaluated to show moderate antimicrobial activity against *Mycobacterium bovis* bacille Calmette–Guérin (BCG) (Liu et al., 2016; Figure 13).

**Figure 14:**
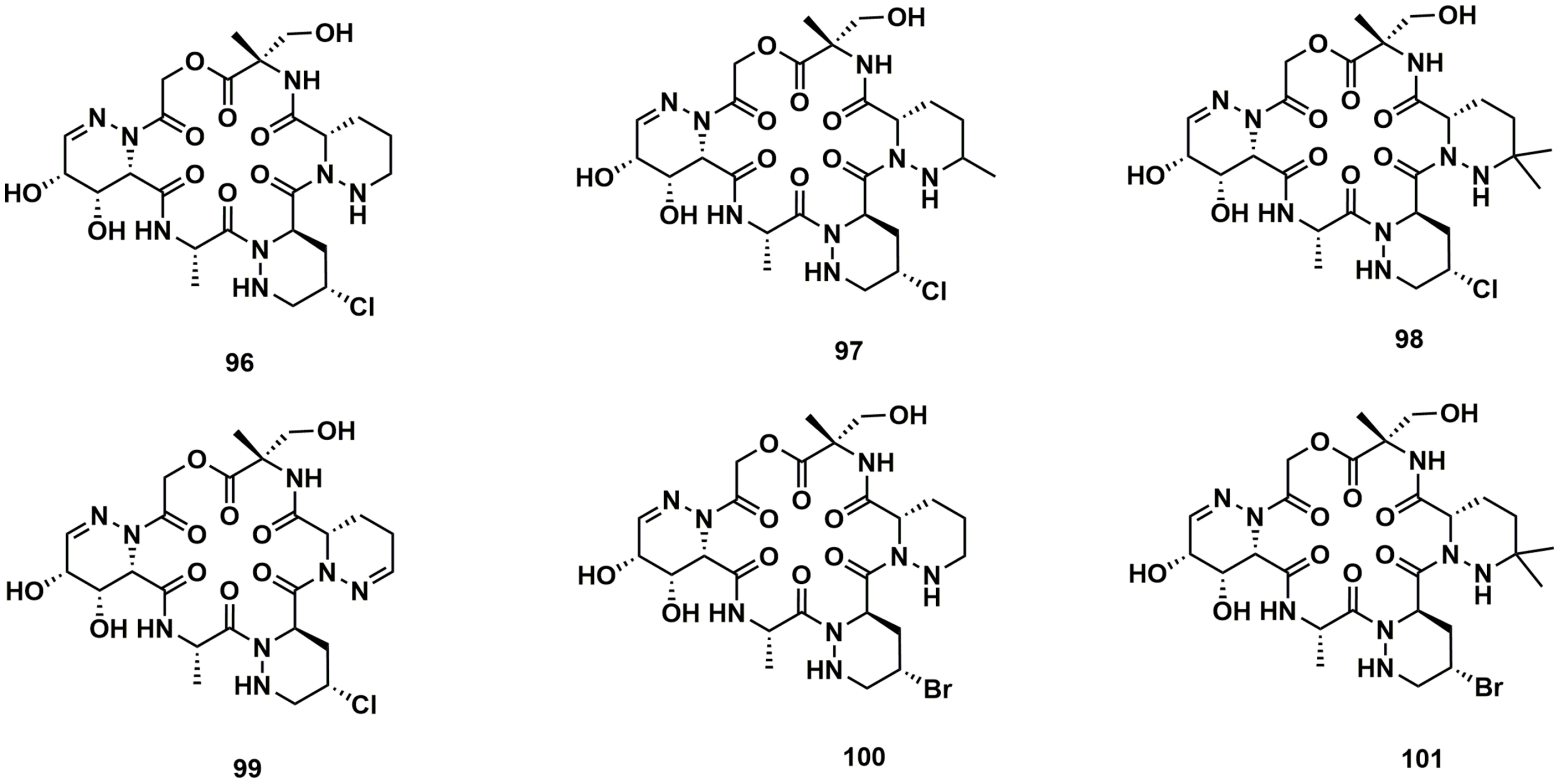
Chemical structures of CDPs **96**–**101**.

A group of cyclodepsipeptides that included piperazic acid, svetamycins A (**96**), B (**97**), C (**98**), D (**99**), *F* (**100**) and G (**101**), were separated from *Streptomyces* sp. DSM14386. With an IC_80_ value of 2 μg/mL, **101**, the strongest antibacterial compound in this group of substances, prevented the development of *Mycobacterium smegmatis* (Dardić et al., 2017; Figure 14).

### Cyclodepsipeptides from other bacteria

**Figure 15:**
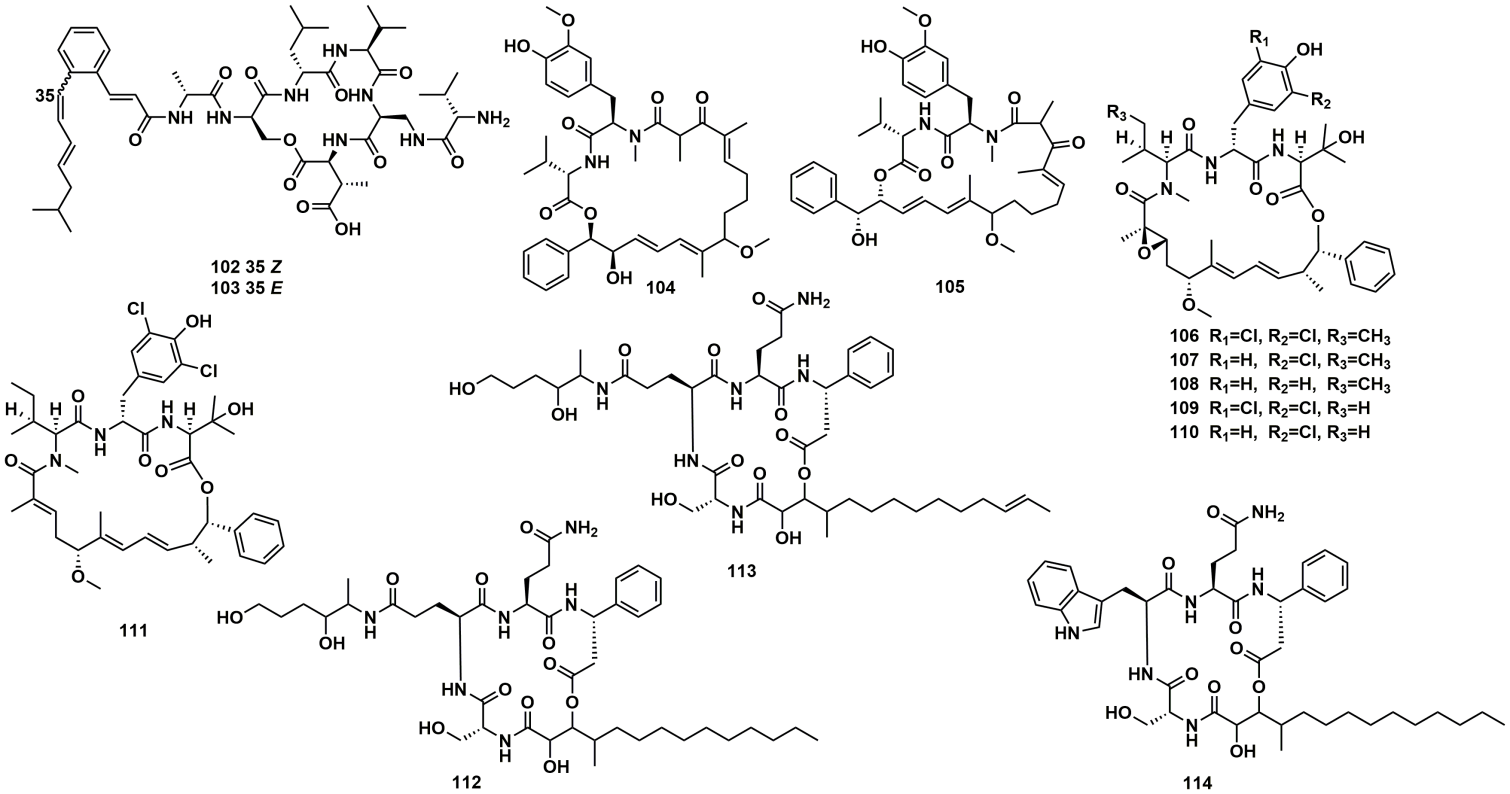
Chemical structures of CDPs **102**–**114**.

Spectroscopic analysis elucidated structures of two new compounds, coprisamides C and D (**102** and **103**), discovered from a dung beetle gut bacterium, *Micromonospora* sp. UTJ3. **102** exhibited a moderate inhibitory effect on the *Mycobacterium tuberculosis* mc^2^ 6,230 strain (Shin et al., 2021). Aetheramides A (**104**), and B (**105**), which had IC_50_ values of 0.015 and 0.018 μM, respectively, showed strong inhibitory effects against HIV-1 (Table 3). Furthermore, **104** displayed cytotoxic activity with an IC_50_ value of 0.11 μM against the human HCT-116 (Plaza et al., 2012; Figure 15). Nannocystin A (**106**) was discovered from a myxobacterial genus, *Nannocystis* sp. The IC_50_ value for compound **106**, which inhibited the growth of *C. albicans*, was 73 nM, indicating a strong antifungal activity. Besides **106**, *Nannocystis* sp. also yielded nannocystin A1 (**107**), nannocystin A0 (**108**), nannocystin B (**109**), nannocystin B1 (**110**) and nannocystin Ax (**111**) (Hoffmann et al., 2015; Krastel et al., 2015). Alveolaride A (**112**), alveolaride B (**113**) and alveolaride C (**114**) were isolated from *Microascus alveolaris* strain PF1466. Alveolaride A (**112**) exhibited a potent inhibitory effect on the plant pathogens *Zymoseptoria tritici, Ustilago maydis,* and *Pyricularia oryzae*. Alveolaride C (**114**) was solely effective toward *U. maydis*, but alveolaride B (**113**) was effective toward both *U. maydis* and *Z. tritici* under *in vitro* conditions (Fotso et al., 2018; Figure 15).

**Table 3:** Other biological activities of CDPs.

Compound	Biological activity	Materials	LD_50_, IC_50_	References
33	Brine shrimp lethal	Brine shrimp	^a^ 2.78 μg/mL	Wang et al. (2020)
36	Antitrypanosomal activity	*Trypanosoma brucei brucei*	^b^ 8.56 μg/mL	Umeyama et al. (2014)
37	Antitrypanosomal activity	*Trypanosoma brucei brucei*	^b^ 8.65 μg/mL	Umeyama et al. (2014)
38	Antitrypanosomal activity	*Trypanosoma brucei brucei*	^b^ 8.63 μg/mL	Umeyama et al. (2014)
48	Antitrypanosomal activity	*Trypanosoma brucei brucei* GUTat3.1	^b^ 0.33 μg/mL	Ganaha et al. (2016)
49	Antitrypanosomal activity	*Trypanosoma brucei brucei* GUTat3.1	^b^ 0.16 μg/mL	Ganaha et al. (2016)
50	Antitrypanosomal activity	*Trypanosoma brucei brucei* GUTat3.1	^b^ 0.061 μg/mL	Ganaha et al. (2016)
52	Superoxide anion production inhibition	Human neutrophils	^b^ 10.00 ± 0.80 μM	Chung et al. (2013)
53	Superoxide anion production inhibition	Human neutrophils	^b^ 10.90 ± 0.59 μM	Chung et al. (2013)
	Elastase release inhibition	Human neutrophils	^b^ 10.01 ± 0.46 μM	
44	Superoxide anion production inhibition	Human neutrophils	^b^ 12.21 ± 0.98 μM	Chung et al. (2013)
	Elastase release inhibition	Human neutrophils	^b^ 12.76 ± 1.00 μM	
55	Superoxide anion production inhibition	Human neutrophils	^b^ 10.09 ± 0.83 μM	Chung et al. (2013)
	Elastase release inhibition	Human neutrophils	^b^ 12.12 ± 0.72 μM	
57	Elastase release inhibition	Human neutrophils	^b^ 15.09 ± 0.28 μM	Chung et al. (2013)
67	Antimalarial activity	*Plasmodium falciparum* K1	^b^ 1.6 μg/mL	Isaka et al. (2014)
68	Antimalarial activity	*Plasmodium falciparum* K1	^b^ 6.4 μg/mL	Isaka et al. (2014)
69	Antimalarial activity	*Plasmodium falciparum* K1	^b^ 1.6 μg/mL	Isaka et al. (2014)
86	Selective chymotrypsin inhibition	Chymotrypsin	^b^ 2.1 μM	Yang et al. (2018)
87	Selective chymotrypsin inhibition	Chymotrypsin	^b^ 1.1 μM	Yang et al. (2018)
104	Antiviral activity	HIV-1	^b^ 0.015 μM	Plaza et al. (2012)
105	Antiviral activity	HIV-1	^b^ 0.018 μM	Plaza et al. (2012)

^a^LD_50_, concentration required for 50% lethality; ^b^IC_50_, concentration required for 50% inhibition.

## Discussions

The chemical structures of cyclodepsipeptides, especially the absolute configurations, were complicated and difficult to determine, different methods were needed. Among them, Marfey’s method, modified Mosher’s method, and X-Ray diffraction analysis besides 1D-NMR and 2D-NMR (Wang et al., 2014; Liu et al., 2020; Wang et al., 2022) were the best choices for determination of the configurations for those compounds. Moreover, the MS–MS fragment analysis was also of great use for judging its sequence of the amino acids (Chung et al., 2013; Liu et al., 2020).

As expected, an abundance of fungi and bacteria-derived cyclodepsipeptides were isolated, and most of them showed significant cytotoxic activities. It was suggested that the cyclic depsipeptide structure was of great importance for the biological activity, because in cytotoxicity assay, the linear homologs of the cyclohexadepsipeptide paecilodepsipeptide A were inactive (Hamann et al., 1996). In addition, the scope of bioactivity of cyclodepsipeptides spanned a range from cytotoxity, and anti-bacterial to anti-malarial activity. Thus, it was suggested that cyclodepsipeptides were desirable chemical species, and could be further applied as leading compounds in drug research.

Although most of the fungi and bacteria have been shown to be a rich source for discovering cyclodepsipeptides, the number of new cyclodepsipeptides was still limited. Conventional isolation method was time-consuming and inefficient, it was necessary to develop a more effective method to explore cyclodepsipeptide candidates. Fortunately, some studies have illustrated that the biosynthesis of cyclodepsipeptides were accomplished nonribosomally by cyclodepsipeptide synthetases (Bills et al., 2014; Du et al., 2014; Aleti et al., 2015; Liu et al., 2015), thus, targeted discovery of cyclodepsipeptides by genomic analysis became possible. By use of targeted isolation methods, such as genome mining as well as molecular networking method (Duncan et al., 2015; Paulo et al., 2019; Wei et al., 2021; Clements-Decker et al., 2022), should be paid more attention in the future in order to obtain those bioactive compounds more efficiently.

In addition, due to the small amount of cyclodepsipeptides from natural products, investigations on *in vivo* effects and on the detailed mechanism of the bioactivities were limited. To solve this problem, sufficient compound material was however required. Facing the problem, total synthesis and heterologous expression of genes or gene clusters in microbial hosts were two better ways, which were keys to access industrially and pharmaceutically relevant compounds in an economically affordable and sustainable manner (Stolze and Kaiser, 2013; Doi, 2014; Roderich et al., 2015).

## Conclusion

In conclusion, this review gave an overview of as many as 114 natural cyclodepsipeptides isolated and identified from fungi and bacteria since 2010, among them, endophytic fungi of plant were the largest group of producers. The review enriched our knowledge about structural features of cyclodepsipeptides and their biological sources.

## Author contributions

S-XL: Data curation, Investigation, Writing – original draft. S-YO-Y: Data curation, Investigation, Writing – original draft. Y-FL: Data curation, Investigation, Writing – original draft. C-LG: Data curation, Investigation, Writing – original draft. S-YD: Data curation, Investigation, Writing – original draft. CL: Investigation, Project administration, Writing – review & editing. T-YY: Investigation, Project administration, Writing – review & editing. Y-HP: Investigation, Project administration, Supervision, Writing – review & editing.

## Funding

The author(s) declare that no financial support was received for the research, authorship, and/or publication of this article.

## Conflict of interest

The authors declare that the research was conducted in the absence of any commercial or financial relationships that could be construed as a potential conflict of interest.

## Publisher’s note

All claims expressed in this article are solely those of the authors and do not necessarily represent those of their affiliated organizations, or those of the publisher, the editors and the reviewers. Any product that may be evaluated in this article, or claim that may be made by its manufacturer, is not guaranteed or endorsed by the publisher.
